# Predictors and Health Consequences of Screen-Time Change During Adolescence—1993 Pelotas (Brazil) Birth Cohort Study

**DOI:** 10.1016/j.jadohealth.2012.06.025

**Published:** 2012-12

**Authors:** Samuel Carvalho Dumith, Leandro Martin Totaro Garcia, Kelly Samara da Silva, Ana Maria Baptista Menezes, Pedro Curi Hallal

**Affiliations:** a Department of Population and Health, Federal University of Rio Grande, Rio Grande, Brazil; b Department of Biological and Health Sciences, Cruzeiro do Sul University, Sao Paulo, Brazil; c Department of Physical Education, Federal University of Santa Catarina, Santa Catarina, Brazil; d Department of Social Medicine, Federal University of Pelotas, Pelotas, Brazil

**Keywords:** Sedentary lifestyle, Leisure activities, Adolescents, Body composition, Adiposity, Cohort studies

## Abstract

**Purpose:**

To investigate screen-time change from early to mid adolescence, its predictors, and its influence on body fat, blood pressure, and leisure-time physical activity.

**Methods:**

We used data from a longitudinal prospective study, conducted among participants of the 1993 Pelotas (Brazil) Birth Cohort Study. At baseline, adolescents were, on average, 11 years old. They were later visited at age 15 years. Screen time was self-reported, accounting for the time spent watching television, playing video games, and using the computer. Several predictors were examined. The effect of screen-time change on some health outcomes was also analyzed.

**Results:**

Screen time increased on average 60 min/d from 11 to 15 years of age, for the 4,218 adolescents studied. The groups that presented the highest increases in screen time were male, wealthiest, those whose mothers had higher education, and adolescents with a history of school failure. There were positive associations between screen-time change and body mass index, skinfold thickness, waist circumference, and leisure-time physical activity at 15 years of age.

**Conclusions:**

Screen time increased from early to mid adolescence. This increment was higher among boys and the wealthiest adolescents. Increases in screen time affected body composition, with negative implications on adiposity.

**Implications and Contribution**

Screen time increased from early to mid adolescence. This rise was higher among boys and the wealthiest adolescents. Increases in screen time affected body composition, with negative implications on adiposity.

Despite the recent accumulated evidence about the increase in overall screen time among adolescents [1,2], little is known about the patterns of change of this behavior throughout adolescence and its health consequences. Studies discussing the tracking of screen time during adolescence to adulthood revealed possible stability [3] or a decline in time spent on these activities [4]. On the other hand, the claim that these behaviors have an independent effect on health is still emerging [5,6]. It is suggested that too much screen time is associated with adverse health behaviors and sociocognitive outcomes in young people [7]. However, answers to some questions are still needed, such as What factors influence the tracking of screen time during adolescence?; What are the health consequences it generates? and Which are the groups most at risk?

Researchers have evaluated the predictive strength of some variables on screen time. The results revealed a positive association between screen time and gender (male), body mass index (BMI), and depression, and an association with non-Caucasian, socioeconomic status, and parental education [8]. However, when only prospective studies are considered, there is insufficient evidence on the determinants of sedentary behavior, including screen time during adolescence [9].

Unlike physical activity, research studies about the determinants of sedentary behavior in children and adolescents are scarce, and there is a consensus among researchers that there is an insufficient accumulation of information [8,9]. Few studies have been conducted in this field; there is a wide variation in the types of sedentary behaviors studied and the limited amount of predictor variables analyzed. In this scenario, it seems necessary to understand the demographic, biological, psychological, behavioral, social, and environmental predictors of screen time.

Identifying the predictors of screen time is also justified by the growing concern that this time may bring detrimental consequences to health. In fact, in the past 10 years, there has been a rapid increase of evidence that sustains this hypothesis, demonstrating a positive association between sedentary behavior, specifically screen time, with all-cause mortality [10] and increased cardiometabolic risk [11].

Therefore, the purpose of this study was to analyze the change in screen time of adolescents from 11 to 15 years of age, its predictors, and its influence on body fat, blood pressure, and leisure-time physical activity in a birth cohort in Southern Brazil.

## Methods

### Study design and population

We used data from a longitudinal prospective study conducted among participants of the 1993 Pelotas (Brazil) Birth Cohort Study. This cohort included all children born in the calendar year of 1993 (N = 5,249) in Pelotas, a city in Southern Brazil with a population of 320,000. In 2004, when they were on average 11 years old (mean = 11.3; standard deviation [SD] = .3), all participants were searched for follow-up, and 4,452 members (87.5%) of the original cohort were traced. In 2008, when they were on average 15 years old (mean = 14.7; SD = .3), all individuals were sought again, and 4,325 were followed up (85.2%). Overall, 4,118 adolescents (81.2% of original cohort) had complete information regarding sedentary behavior for both waves (11 and 15 years). There was no difference in terms of sedentary behavior levels (min/d) between those who were interviewed in both periods and those who were not located in the last survey in 2008. Moreover, the profile of individuals included in this study and the original cohort was similar in terms of socioeconomic, demographic, and anthropometric variables.

Average follow-up duration was 3.4 years (SD = .2), ranging from 2.8 to 4.0 years. Detailed information about the cohort methodology and previous follow-ups is published elsewhere [12]. The study protocol was approved by the Ethics Committee of the Medical School from the Federal University of Pelotas, and the parents or guardians of all participants signed a written informed consent.

### Logistics and instrument

Each data collection period (2004 and 2008) lasted approximately 8 months. The first one was carried out from July 2004 to March 2005, whereas the second wave extended from January to August 2008. The methodology used in both surveys was the same. Data were collected through face-to-face home interviews by trained interviewers. A standardized and pretested questionnaire was used. One questionnaire was administered to mothers (or guardians) and another, with different questions, to the adolescents. Measurements of weight, height, and subscapular and triceps skinfold were collected in both waves. Skinfolds were measured using a Cescorf scientific caliper (Cescorf, Porto Alegre, Brazil) following the recommendations of Lohman et al [13]. Weight and height were obtained using a SECA digital scale (SECA, Birmingham, UK) with 100-g precision and an aluminum stadiometer with 1-mm precision, respectively. When adolescents were 15 years old, they had their waist circumference and blood pressure measured twice by trained technicians. Blood pressure was measured by a wrist digital (OMRON HEM 629, Beijing, China), and a correction equation was used [14].

For quality control purposes, 30% of the participants were reinterviewed by two study supervisors (10% in person and 20% by telephone calls) using a short questionnaire. Additionally, all questionnaires were checked for completeness and consistency by study supervisors.

### Screen time

Screen time was collected through face-to-face interviews with the adolescents. The instrument included questions on whether the adolescent watched television (TV), played video games, and used the computer. The translated questions were (1) “How much time do you watch TV?”; (2) “How much time do you play video game?”; (3) “How much time do you use the computer?”. Interviewers were trained to identify possible overlap (e.g., if the same time is reported in both situations) and ask the respondent to choose the appropriate answer in such cases.

The mean time spent in front of each of these electronic media (in a typical week) was noted separately for weekdays and weekends. The outcomes were constructed by adding the weighted mean screen time (TV + video game + computer), assigning the weight 5 to weekdays, 2 to weekends, and dividing the result by 7 to obtain the mean time in minutes per day. Screen-time change between 11 and 15 years of age was calculated by subtracting the time (min/d) at age 15 years by the respective time at age 11 years.

### Predictors

The variables included as possible predictors of screen-time change were sex; self-reported skin color (white, mixed, black); socioeconomic level at baseline, generated by principal component analysis of 19 assets index in the household [12] and categorized into tertiles (poorest, intermediate, wealthiest); maternal schooling (0–4, 5–8, 9–11, ≥12 years), adolescent failure in school (no, yes); amount of time spent outdoors in comparison with peers, self-reported by the adolescent at baseline (mostly indoors, mostly outdoors); perception of fear of living in the neighborhood at baseline (no, yes); relationship with parents (fair, excellent); BMI status at age 11 years, based on objective measurement of weight and height, and classified according to the cut-off points proposed by the World Health Organization [15] (underweight or normal, overweight, obese); leisure-time physical activity status at age 11 years, self-reported and categorized according to the cut-off point of 420 min/wk [16]; fat consumption based on the Block questionnaire [17]; and biological maturation at age 15 years, self-reported based on Tanner's stages [18] (least developed, stages 1, 2, and 3, and most developed, stages 4 and 5).

### Outcomes

We studied the effect of screen-time change on several outcomes at age 15 years: BMI (kg/m^2^), sum of subscapular and tricipital skinfold thickness (mm), waist circumference (cm), leisure-time physical activity (min/wk), and systolic and diastolic blood pressure (mm Hg).

### Statistical analyses

Data were double entered by two data-entry clerks and then analyzed in Stata 11 (StataCorp, College Station, TX). Screen-time change (min/d) was analyzed by multiple linear regression analyses, after testing for normality of the distribution. Two models were run: model 1, with adjustment for baseline screen-time level (min/d at 11 years); and model 2, with further adjustment for the covariates. The adjusted analyses followed a three-level hierarchical model. On the first level, adjustments were made for sex, skin color, socioeconomic level, and maternal schooling; in the second, adolescent failure at school, time spent outdoors compared with peers, fear of living in the neighborhood, and relationship with parents, all of them collected at the baseline, were taken into account; in the third, variables adjusted for included BMI, physical activity and diet at baseline, and biological maturation. Each variable was adjusted for those of the same or upper levels. Results were not stratified by sex because there was no interaction with this variable. Statistical tests were two-sided, with a significance level of 5%.

## Results

Overall, 4,218 adolescents were followed up from age 11 to 15 years. Of these, 51% were females, 66% had white skin color, 37% had at least one failure in school, 74% spent time mostly indoors compared with their peers, 15% reported fear of living in the neighborhood, 65% reported having an excellent relationship with their parents, 31% were overweight or obese, 72% were physically inactive (<420 min/wk), 37% ate a diet rich in fat, and 45% were classified in higher stages of pubertal development.

Table 1 shows that the groups that presented the highest increase in screen-time change were males, those of white skin color, wealthiest, those whose mother achieved higher education, adolescents with no failure in school, those with a better relationship with their parents, and those most biologically developed. Time spent outdoors, fear of living in the neighborhood, BMI, physical activity status, and diet at baseline did not present an association with screen-time change. After adjustments for screen time at baseline and for possible confounders, skin color, relationship with parents, and biological maturation lost its association with the outcome (Table 2).

The outcomes of screen-time change are shown in Table 3. Among adolescents who increased their screen time, an increase in their BMI, skinfold thickness, waist circumference, and leisure-time physical activity at age 15 years was found, showing a positive association of screen-time change with adiposity and physical activity during adolescence. No association was verified with blood pressure (Table 3).

## Discussion

This study aimed to identify the predictors and health consequences of screen-time changes from 11 to 15 years of age in a birth cohort from Southern Brazil. To our best knowledge, this is the first study in Latin America to address this subject and one of the few in the international literature. We investigated both the predictors and outcomes of screen-time change in adolescents.

Some limitations of our study are the self-reported information of screen time and the short follow-up period. Another possible limitation was to have analyzed change in time spent watching TV, playing video game, and using the computer as a composite measure. Nonetheless, stratifying the analyses for each component revealed that most associations were observed in the same direction, although the strongest associations have seen a change in computer time. An issue to be considered when interpreting our findings is that adolescent boys and girls are growing at rapid rates from age 11 to 15 years. This might influence physical activity practice, and as a consequence, screen time as well.

A systematic review showed that screen time tracks from childhood and adolescence, thus suggesting that interventions should start as early as possible [3]. Our study focused on some key behaviors such as watching TV, using computer, and playing video games. We found an increase of 60 minutes (95% confidence interval [CI]: 53.0–66.0) in the overall screen time from age 11 to 15 years. Computer use increased (82 minutes; 95% CI: 78.0–85.0), TV viewing declined (−12.3 min; 95% CI:−16.8 to −7.8), and no change occurred in time spent playing video games (.4 minutes; 95% CI: −3.0 to 2.4).

Previous studies found that watching TV/videos/DVDs and using a computer for fun were the most popular sedentary behavior among students, corresponding with more than one-half of all sedentary time spent by Australian adolescents [19]. Consistent with previous studies [1,4,20,21], our study found that boys reported more hours of screen time than girls. In American adolescents, there was no difference in mean time of watching TV/video between sexes. However, boys spent more time playing computer games than girls, whereas girls spent more time sitting and listening to music and talking on the telephone than boys [22]. In Spanish children, males also spent more time playing games—consoles—and engaged in more time spent in all screen-viewing behaviors than females [23].

Our findings showed greater screen time among adolescents most biologically matured compared with those in previous stages. However, when adjusted for all variables, biological maturation lost its association, suggesting that other factors might influence this relationship. Researchers have shown that more screen time occurs in British adolescents in greater biological development stages [24]. Indeed, with the advancement of puberty stages, adolescents may feel more attracted to new and complex technologies.

Screen time in our study was greater in adolescents of white skin color, higher maternal education, and improved assets index. Apart from skin color, these variables remained significant in the final model. Another study also found lower screen time among black adolescents compared with white teens [4]. However, a British study revealed that black students accumulated more screen time than their white counterparts [25]. Recently, a review of studies revealed an inverse association among sedentary behaviors and ethnicity (Caucasian), socioeconomic status, and parental education in adolescents [8,26]. Conversely, in a systematic review involving only prospective studies, authors found insufficient evidence for the socioeconomic determinants of sedentary behavior [9].

This scenario shows that these associations are still inconclusive and inconsistent, suggesting that predictors of screen time might be influenced by particular characteristics of the studied population, advances of and access to technology, and the local culture of technology consumption in each country. Nevertheless, more evidence involving prospective studies is extremely relevant to understanding predictors of screen time and the reasons why some studies have found inverse association [8,26], no association [9,19], or direct associations (e.g., our study). A survey using data from countries participating in the Health Behaviour in School-Aged Children study found that the engagement in screen activities may have different meaning in different cultures and may be influenced by behavioral traditions [20], which partially may explain these differences in findings, reinforcing our suppositions.

Our findings indicated that overall screen time was lower in adolescents with poor school performance in all tested models. Several studies with babies and children demonstrated that high exposure to TV could result in poorer cognitive development, short-term memory, attention, language skills, and academic achievement [7,27]. However, as pointed out by Roberts et al [28], media is becoming more integrated into the lives of young people, and its impact on academic performance could be attenuating or changing direction in the past decades. It is possible that this phenomenon is associated with computer use—the behavior with the most important increase in our study—for academic purposes. Today, many screen electronic components might be used as tools to help students with homework and diverse school content. Adolescents with greater academic interest may seek access to computers, particularly to have greater availability to information, didactic materials, and others facilitators of the learning process.

In the present study, screen time was greater in adolescents with excellent relationships with parents in crude analyses and when adjusted to screen time at baseline. However, in the final model, an association between relationship with parents and increase in screen time was not found. Perhaps, the enjoyable family environment may increase the time adolescents spend at home; thus, a good relationship with parents might exert stronger influence on adolescent screen time. A Chinese study identified that children who often watched TV with their parents were 2.3 times more likely to spend more screen time than those who seldom watched TV with their parents [1]. In addition, findings with Spanish children suggest that parental screen-viewing rules and family co-viewing practices appeared to be predictors of screen viewing [29]. The Health Behaviour in School-Aged Children data reported a positive association between screen-based media sedentary behaviors and quality of peer relationships, in all regions studied. In contrast, a negative relationship with quality of family relationships was found [20]. In our study, this association did not persist after adjustment for confounders; one explanation is that the involvement of parents in the types of screen activities practiced by adolescents was not asked. Possibly, the engagement of parents in these activities may directly influence overall screen time.

In our study, teens who perceived spending more time indoors compared with peers did not have more screen time than those who perceived spending more time outdoors. Similarly, the feeling of safety about the neighborhood where they live did not affect screen time. Some possible explanations for these findings are that in Brazil, it is common to find commercial enterprises with easy access to screen technologies in neighborhoods regardless of security, such as Internet cafes, video gaming facilities, and other options. The fact that time spent indoors or outdoors was not a strong predictor of screen time could be because screen activities might be found in both environments; young people have access to video games and laptop computers and may transport them to different locations, access the Internet anytime, or even attend houses specializing in video games or other electronics. In Chinese youth, the odds of high screen time were strongly associated with having access to Internet at Internet cafes or at home [1]. Few studies have assessed the relationship of these predictors with screen time, and more evidence is necessary.

Our study found no evidence of prediction of change in screen time related to physical activity engagement at age 11 years. Literature consistently points out that screen time has no or just moderate associations with leisure-time physical activity, indicating that they are two different and independent behaviors [11,30].

Alternatively, we found a positive association between change in screen time and leisure-time physical activity at age 15 years, owing mainly to video game playing time. Other researchers have found a relationship between video game playing specifically and leisure-time physical activity [2,31,32]. However, the explanation is not clear. A hypothesis is that video games featuring sports may encourage playing sports in real life. As pointed out by Marshall et al [32], it is possible that the relationship between screen time and physical activity is multifaceted and dependent of the behavior analyzed.

In the case of BMI, we found the same pattern as seen with physical activity engagement. We did not find evidence to suggest that change in screen time predicts BMI at baseline in the final model. However, a positive association between the change in screen time from age 11 to 15 years and BMI was found. Our results are consistent with other recent longitudinal studies [33] and literature reviews [6,7], although there are still many controversies [5,34]. Some hypotheses try to explain the relationship between screen time and body fat (1) obesity itself increases screen time; (2) displacement of physical activities with screen time, resulting in a reduction in total energy expenditure; (3) reduced resting energy expenditure during screen time; and (4) higher consumption of unhealthy food (such as sweets, cakes, and fast foods), especially during TV viewing [35].

In terms of blood pressure, our study did not find evidence of association, except between change in TV viewing and diastolic blood pressure (data not shown). In a recent review, Tremblay et al [6] found that four of nine cross-sectional studies observed a positive association between screen time and blood pressure. Only two longitudinal studies are available in the literature about this subject: one did not find any association [36], and the other did not find association with overall screen time, TV viewing, and computer use, but found a relationship with video game playing [37].

In summary, screen time increased from early to mid adolescence. This rise was higher among boys and the wealthiest adolescents. Increases in screen time from age 11 to 15 years were related to body composition at age 15 years, with a negative implication on adiposity. Further studies are needed to expand the body of evidence on predictors and health consequences of screen-time change among adolescents.

## Figures and Tables

**Table 1:** Descriptive analyses of the factors associated with screen-time change (min/d) from age 11 to 15 years—1993 Pelotas (Brazil) Birth Cohort Study (N = 4,118)

Variable	n	Screen time at baseline (min/d)	Mean change (min/d)	*p* value
Sex				<.001
Male	2,054	265	74	
Female	2,164	248	46	
Skin color				.03
White	2,692	263	67	
Mixed	602	241	50	
Black	765	242	46	
Assets index				<.001
Poorest	1,352	238	29	
Intermediate	1,363	252	60	
Wealthiest	1,360	282	95	
Maternal schooling (years)				<.001
0–4	1,067	239	20	
5–8	1,819	253	55	
9–11	903	272	91	
≥12	405	287	115	
Adolescent failure in school				<.001
No	2,643	270	77	
Yes	1,552	235	30	
Time spent outside compared with peers				.25
Mostly outdoor	1,096	251	67	
Mostly indoor	3,111	259	58	
Fear of living in the neighborhood				.22
No	3,538	254	62	
Yes	671	270	51	
Relationship with parents				.02
Fair	1,453	250	50	
Excellent	2,666	262	67	
BMI at age 11 years				.18
Normal	2,905	249	56	
Overweight	852	269	69	
Obese	456	277	69	
Active at age 11 years				.89
No	2,949	254	60	
Yes	1,151	261	59	
Diet rich in fat				.11
No	2,667	249	56	
Yes	1,547	269	67	
Sexual maturation				.004
Least	1,849	252	49	
Most	1,525	270	71	
Total	4,218	256	60	—

BMI = body mass index.

**Table 2:** Crude and adjusted analyses for the factors associated with screen-time change (min/d) from age 11 to 15 years—1993 Pelotas (Brazil) Birth Cohort Study (N = 4,118)

Variable	Unadjusted	Model 1^a^	Model 2^b^
	Beta (95% CI)	Beta (95% CI)	Beta (95% CI)
Sex			
Male	0	0	0
Female	−29 (−42, −15)	−41 (−52, −30)	−41 (−52, −30)
Skin color			
White	0	0	0
Mixed	−16 (−34, 1)	−33 (−47, −18)	−8 (−23, 7)
Black	−22 (−41, −2)	−38 (−54, −22)	−13 (−30, 4)
Assets index			
Poorest	0	0	0
Intermediate	32 (15, 47)	42 (28, 55)	32 (18, 46)
Wealthiest	66 (49, 82)	100 (86, 113)	63 (47, 80)
Maternal schooling (years)			
0–4	0	0	0
5–8	35 (19, 51)	46 (33, 60)	35 (20, 49)
9–11	71 (51, 90)	97 (81, 113)	68 (50, 85)
≥12	95 (70, 120)	132 (112, 153)	87 (64, 111)
Adolescent failure in school			
No	0	0	0
Yes	−47 (−61, −34)	−75 (−86, −63)	−51 (−64, −38)
Time spent outside compared with peers			
Mostly outdoor	0	0	0
Mostly indoor	−9 (−24, 6)	−3 (−16, 10)	−1 (−13, 12)
Fear of living in the neighborhood			
No	0	0	0
Yes	−11 (−30, 7)	0 (−15, 15)	9 (−7, 24)
Relationship with parents			
Fair	0	0	0
Excellent	16 (2, 30)	25 (13, 37)	4 (−8, 16)
BMI at age 11 years			
Normal	0	0	0
Overweight	13 (−3, 30)	29 (15, 43)	8 (−8, 23)
Obese	13 (−8, 35)	34 (16, 53)	11 (−9, 32)
Active at age 11 years			
No	0	0	0
Yes	−1 (−15, 13)	5 (−7, 16)	−5 (−17, 8)
Diet rich in fat			
No	0	0	0
Yes	11 (−3, 25)	26 (15, 38)	11 (−3, 23)
Sexual maturation			
Least	0	0	0
Most	22 (7, 36)	35 (22, 47)	5 (−9, 18)

CI = confidence interval.

a Model 1: Adjusted for screen time (min/d) at baseline.

b Model 2: Adjusted for model 1 plus variables in the table, according to a conceptual model.

**Table 3:** Linear regression analyses on consequences of screen-time change (hr/d) from age 11 to 15 years—1993 Pelotas (Brazil) Birth Cohort Study (N = 4,118)

Outcome	Unadjusted	Model 1^a^	Model 2^b^
	Beta (95% CI)	Beta (95% CI)	Beta (95% CI)
BMI (kg/m^2^)	.02 (−.02, .05)	.06 (.02, .10)	.06 (.01, .10)
Sum of skinfolds (mm)	.001 (−.1, .1)	.09 (−.05, .22)	.2 (.1, .3)
Waist circumference (cm)	.1 (.01, .2)	.2 (.1, .3)	.1 (.04, .2)
Leisure-time PA (min/wk)	8 (4, 11)	11 (7, 16)	8 (3, 11)
Systolic BP (mm Hg)	.04 (−.04, .13)	.16 (.06, .26)	.04 (−.06, .14)
Diastolic BP (mm Hg)	−.04 (−.09, .01)	−.04 (−.10, .02)	.04 (−.02, .11)

BP = blood pressure.

a Model 1: Adjusted for screen time (min/d) at baseline.

b Model 2: Adjusted for model 1 plus sex, skin color, assets index, and maternal schooling.
